# Hyperhomocysteinemia: A Rare Cause of Isolated Ischemic Abducens Nerve Palsy in Young Adults

**DOI:** 10.22599/bioj.558

**Published:** 2026-11-12

**Authors:** Dipti Shikha Kashyap, Pramod Kumar Pandey, Anupam Singh, Barun Kumar

**Affiliations:** 1 Department of Ophthalmology, ESIC Medical College and Hospital, Faridabad, Haryana, India; 2 AIIMS Rishikesh, India; 3 Department of Cardiology, AIIMS Rishikesh, Uttarakhand, India

**Keywords:** Isolated cranial nerve palsy, abducens nerve palsy, hyperhomocysteinemia, ischemic cranial nerve palsy

## Abstract

Ischemic ocular motor nerve palsies typically manifest as an isolated unilateral event in patients over the age of 45 years who have known risk factors, such as hypertension, diabetes mellitus, and dyslipidemia. Blood dyscrasias, hypercoagulable states, sickle cell disease, and congenital cardiac disease are likely culprits in younger age groups. Hyperhomocysteinemia is an unusual presentation of ischemic cranial nerve palsy, but it has been linked to thromboembolic states due to its propensity to damage the vascular endothelium lining. We report six young patients (age 18–45 years) with isolated unilateral abducens nerve palsy, who presented with an acute onset of painless uncrossed diplopia. Elevated homocysteine level of >40 μmol/L (mean value of 51.78 µmol/L) was the only biochemical abnormality detected. There was an uneventful and almost complete recovery in all six cases, over a period of three months after supplementation with vitamins B6, B12, and folic acid. Therefore, serum homocysteine should be investigated in every young patient presenting with an isolated ischemic cranial nerve palsy.

## Introduction

The elevated total homocysteine level has garnered attention as a risk factor for arteriosclerotic vascular events in recent years (Pianka *et al*., 2000; Sachdeva *et al*., 2013). It has been reported as an independent risk factor for ocular vaso-occlusive events, such as nonarteritic ischemic optic neuropathy (NAION), central retinal artery occlusion (CRAO), and central retinal vein occlusion (CRVO), especially in patients of a younger age (Pianka *et al*., 2000). Homocysteine is an intermediary metabolite in the methionine metabolic pathway, and it has been demonstrated that even mildly increased levels (>50 µmol/L) facilitate thrombus formation and increase the risk of arterial occlusion.

Isolated ischemic abducens nerve palsy is the predominant form of paralytic strabismus observed in middle-aged (20–40 years) and older individuals (>40 years) with established risk factors, including diabetes mellitus, hypertension, dyslipidemia, and carotid artery disease. However, isolated abducens nerve palsy can be idiopathic or due to head trauma, intracranial space-occupying lesions, vascular diseases, inflammatory conditions, or demyelinating disorders in young patients (<50 years) (Pianka *et al*., 2000). Hyperhomocysteinemia has been reported as an independent risk factor for systemic, ocular vaso-occlusive disorders, and ischemic nerve palsies, especially in young patients (Pianka *et al*., 2000; Sachdeva *et al*., 2013; Sowbhagya *et al*., 2015). In this case series, we describe six patients (younger than 45 years) with isolated abducens nerve palsy where hyperhomocysteinemia was the sole risk factor.

## Methods

This retrospective case series adhered to the Declaration of Helsinki, included six patients (younger than 45 years) who presented with unilateral ischemic abducens nerve palsy between June 2024 and May 2025. Informed written consent was obtained from all patients. All six patients were investigated for other predisposing factors: trauma, diabetes, hypertension, dyslipidemia, collagen vascular disorders, drug intake, fever, surgical intervention, or any other systemic illness. Serum homocysteine, folic acid, and vitamin B12 levels were investigated, as none of them had any obvious history of ocular trauma, recent surgical or medical intervention, or presence of other risk factors like hypertension, dyslipidemia, and diabetes. All patients underwent a detailed ophthalmic work-up, including best-corrected visual acuity (BCVA), pupillary reaction, non-contact tonometry, and slit lamp examination for anterior and posterior segment details. A thorough orthoptic evaluation was performed to confirm the diagnosis of paralytic etiology of ocular motility limitation, including four steps (Mazow, 1978): evaluation of versions and monocular non-forced ductions, qualitative assessment of saccadic eye movements by observation, forced ductions, and generated forced testing. For the measurement of primary position deviation at near and distance, the alternate prism cover test (APCT) was performed using loose prisms. Table 1 summarizes the clinical and demographic details of the cases.

**Table 1:** Clinical and investigation details of the cases.


AGE (YEARS)	GENDER	DURATION	AFFECTED EYE	DEVIATION AT DISTANCE/MOTILITY	SERUM HOMOCYSTEINE LEVEL AT INITIAL VISIT (NORMAL RANGE: 5.00–20.00 μmol/L)	SERUM FOLIC ACID AND VITAMIN B12 LEVELS (VALUES 5.3 ng/mL AND 211 pg/mL, RESPECTIVELY, WERE CONSIDERED LOW)	SERUM HOMOCYSTEINE LEVEL IN μmol/L (FOLLOW-UP AFTER THREE MONTHS OF TREATMENT)	DEVIATION AT DISTANCE/MOTILITY AFTER THREE MONTHS OF FOLLOW-UP

26	F	10 days	OS	20 PD, –2 abduction	51.25	Folic acid: 4 ng/mL B12: 175 pg/mL	19.67	0 PD, 0 abduction

18	M	7 days	OD	30 PD, –3 abduction	62.10	Folic acid: 3.9 ng/mL B12: 191 pg/mL	15.04	4 PD of esophoria, –0.5 abduction

41	F	8 days	OS	20 PD, –2 abduction	48.00	Folic acid: 4.5 ng/mL B12: 189 pg/mL	19.00	0 PD, 0 abduction

42	M	10 days	OS	22 PD, –2 abduction	38.00	Folic acid: 4.9 ng/mL B12: 180 pg/mL	18.00	0 PD, 0 abduction

45	M	10 days	OD	18 PD, –2	46.20	Folic acid: 4.5 ng/mL B12: 110 pg/mL	19.50	0 PD, 0 abduction

38	M	15 days	OD	18 PD, –2 abduction	65.14	Folic acid: 4.6 ng/mL B12: 190 pg/mL	22.20	0 PD, 0 abduction


## Results

The mean age of the cases was 35 years (range: 18–45 years). Four patients were male, and two were female. Horizontal diplopia was the presenting complaint in all patients, with a mean duration of onset of 10 days (range: 7–15 days). All of them had BCVA of 6/6 (Snellen) in each eye. Abduction limitation ranged from –3 to –2. (Figures 1C and 2A). Mean primary position esotropia at distance was 21.3 prism diopters (PD) (range: 18–30 PD). Qualitative saccadic velocity analysis by clinical observation showed poor amplitude and speed. The forced duction test for the medial rectus muscle was found to be negative, and the force generation test was weakly positive for the lateral rectus in all patients. The anterior and posterior segment examination was unremarkable. All patients underwent contrast-enhanced magnetic resonance imaging (MRI) of the brain, which was normal. All routine blood investigations, including complete blood count, erythrocyte sedimentation rate (ESR), C-reactive protein, lipid profile, and blood glucose levels, were within normal limits. The blood pressure, electrocardiogram, echocardiogram, and carotid Doppler were also unremarkable. There was no history of any thromboembolic episodes in the past, and clinical features were suggestive of isolated unilateral abducens nerve palsy. The serum homocysteine level was elevated in all patients, with a mean value of 51.78 µmol/L (range: 38–65.14 µmol/L; normal range: 3.9–13.9 µmol/L, as determined by the chemiluminescence method) (Kalita *et al*., 2009). Serum folic acid (mean: 4.4 ng/mL, range: 3.9–4.9 ng/mL) and vitamin B12 (mean: 172.5 pg/mL, range: 110–191 pg/mL) levels were found below the normal value (any value lower than 5.3 ng/mL for folic acid and 211 pg/mL for vitamin B12 is considered low) (Kalita *et al*., 2009).

**Figure 1:**
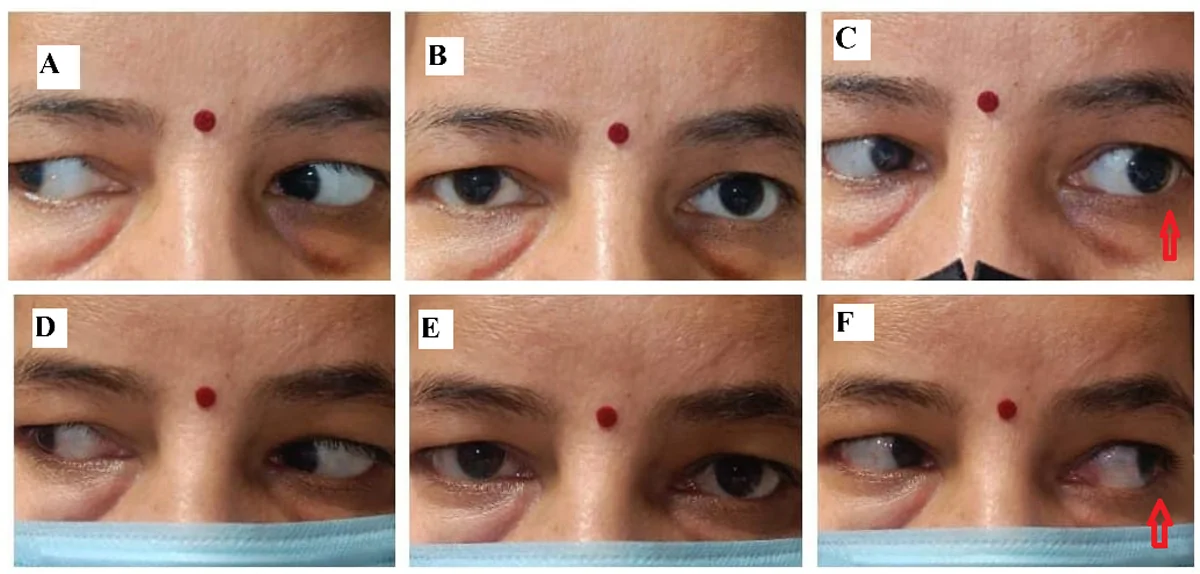
Clinical photograph of patient 1 showing abduction limitation in OS (red arrow, **C**), which improved over three months of follow-up (red arrow, **F**).

**Figure 2:**
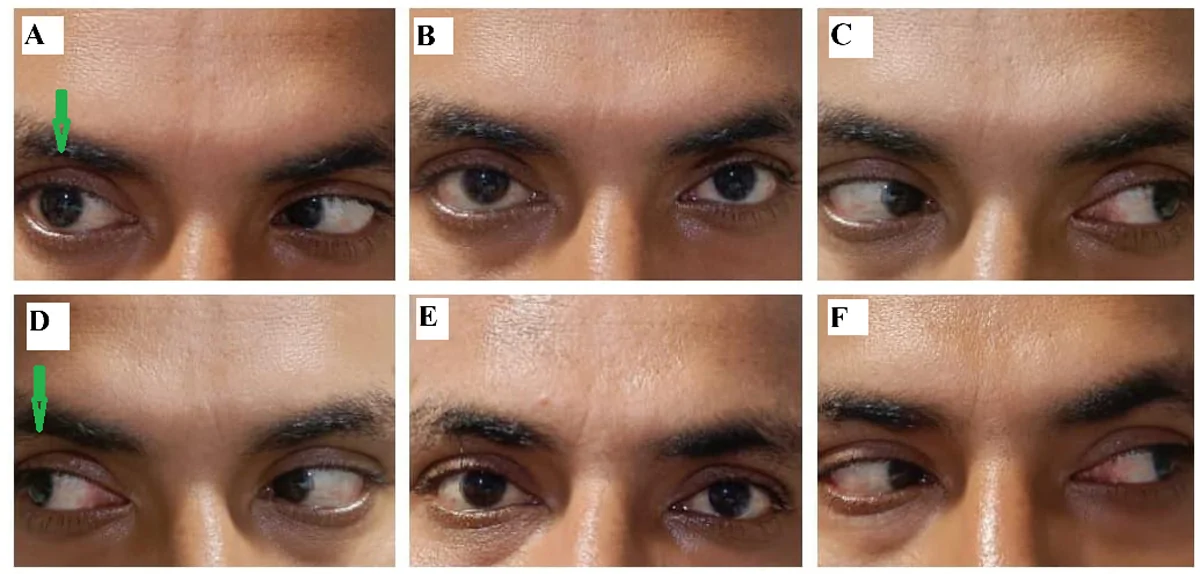
Clinical photograph of patient 2 showing abduction limitation in OD (green arrow, **A**), which improved over three months of follow-up (green arrow, **C**).

In view of the raised serum homocysteine and low serum folic acid and vitamin B12, the final diagnosis in all cases was isolated ischemic abducens palsy due to hyperhomocysteinemia. All six were advised to use alternate patching to occlude diplopia and to take vitamin B6 (250 mg), vitamin B12 (1,500 μg), and folic acid (5 mg) supplements. There was complete resolution of orthophoria in primary gaze and complete recovery of abduction limitation in five patients. One patient had a mild residual abducens nerve palsy, with esophoria of 4 PD at distance and –0.5 residual limitation on abduction after a recovery period of three months. In all six patients, homocysteine levels were reduced to lower values (mean: 18.90 µmol/L, range: 15.04–22.20 µmol/L) over a period of three months (Figures 1F and 2C).

## Discussion

In this case series, we report six cases of ischemic isolated abducens nerve palsy in young adults (aged 18–45 years) who underwent comprehensive ophthalmic work-up and systemic investigations, including neuroimaging. The underlying etiology in all cases was raised serum homocysteine and low serum folic acid and vitamin B12, which led to the final diagnosis of isolated ischemic abducens palsy due to hyperhomocysteinemia. All patients were managed conservatively with alternate patching and vitamin B6 (250 mg), vitamin B12 (1,500 μg), and folic acid (5 mg) supplements and showed complete resolution of symptoms after three months of follow-up.

Moderately elevated serum homocysteine levels (>50 μmol/L) enhance the risk of arterial occlusion and promote the formation of thrombus (Boushey *et al*., 1995). Particularly in younger people, hyperhomocysteinemia has been found to be an independent risk factor for ocular vaso-occlusive disorders like NAION, CRAO, CRVO (Pianka *et al*., 2000), and ischemic palsies in patients (Sachdeva *et al*., 2013; Sowbhagya *et al*., 2015).

Southeast Asians and Indians have been found to have elevated homocysteine levels, which may be due to genetic predisposition, extended high-temperature cooking techniques (Kalita *et al*., 2009), or inadequate dietary intake of folic acid and vitamin B12. Homocysteine metabolism is controlled by acquired cofactors such as pyridoxine, vitamin B12, and folic acid, as well as by genetic variants such as MTHFR, methionine synthase, and cystathionine beta synthase (Brustolin, Giugliani and Félix, 2010; Klee, 2000). It has been found that large- and small-vessel disease, as well as more severe stroke, can be caused by hyperhomocysteinemia. In 2009, Kalita *et al*. reported that hyperhomocysteinemia is seen in both healthy volunteers and 60.6% of ischemic stroke patients in northern India.

Isolated abducens nerve palsy is the most common form of paralytic strabismus in young individuals (<50 years) that may be idiopathic or result from head trauma, intracranial space-occupying lesions, vascular diseases, inflammatory conditions, or demyelinating disorders (Pianka *et al*., 2000). Recently, in younger individuals, hyperhomocysteinemia has been identified as an independent risk factor for ischemic nerve palsies (Sachdeva *et al*., 2013), ocular vaso-occlusive disorders (Pianka *et al*., 2000), and systemic conditions (Sowbhagya *et al*., 2015). A 5 µmol/L rise in homocysteine has been linked to a 60% higher risk of vaso-occlusive diseases in men and an 80% higher risk in women, with an odds ratio of 1.4–1.7 (Boushey *et al*., 1995). The patients in our series were young, with a mean age of 35 years (range: 18–45 years), consistent with earlier studies. Similar to the findings reported by Sachdeva *et al*. (2013), all patients in our case series presented with isolated abducens nerve palsy and had hyperhomocysteinemia as their only known risk factor.

Abducens nerve involvement in these cases could be related to microvasculopathy-induced injury, as seen in diabetic individuals with abducens palsy. Hyperhomocysteinemia represents a possibly modifiable risk factor. Daily dietary supplementation with 250 mg of vitamin B6, 5 mg of folic acid, and 1,500 µg of vitamin B12 has been shown to reduce homocysteine levels and promote spontaneous recovery from nerve palsy, as observed in our case series. Isolated ischemic abducens nerve palsy in young patients due to hyperhomocysteinemia has been reported by Sachdeva *et al*. (2013), in their retrospective case series. However, reasonable explanations for the predilection of the abducens nerve or for young individuals in hyperhomocysteinemia are not well established and warrant additional study. It is possible that some patients are prone to thromboembolic episodes, especially during general anesthesia; proper diagnosis of such cases is of paramount importance before planning for surgical intervention, as they respond very well to dietary supplementation only. Additionally, there is a possibility that routine monitoring of blood homocysteine levels in these patients may aid in the prevention of subsequent thromboembolic incidents. The small sample size of our retrospective series is a drawback, which limits the conclusions that can be made. Based on our findings in six cases, we recommend evaluating homocysteine levels in cases of suspected ischemic cranial nerve palsy before proceeding with neuroimaging. These six patients improved quickly with conservative therapy only, which could eliminate unnecessary and costly clinical evaluations.

## References

[1] Boushey, C.J., Beresford, S.A., Omenn, G.S. and Motulsky, A.G. (1995) ‘A quantitative assessment of plasma homocysteine as a risk factor for vascular disease. Probable benefits of increasing folic acid intakes’, JAMA, 274, pp. 1049–1057. Available at: 10.1001/jama.1995.03530130055028 7563456

[2] Brustolin, S., Giugliani, R. and Félix, T.M. (2010) ‘Genetics of homocysteine metabolism and associated disorders’, Brazilian Journal of Medical Biological Research, 43(1), pp. 1–7. Available at: 10.1590/S0100-879X2009007500021 PMC3078648 19967264

[3] Kalita, J., Kumar, G., Bansal, V. and Misra, U.K. (2009) ‘Relationship of homocysteine with other risk factors and outcome of ischemic stroke’, Clinical Neurology and Neurosurgery, 111, pp. 364–367. Available at: 10.1016/j.clineuro.2008.12.010 19185985

[4] Klee, G.G. (2000) ‘Cobalamin and folate evaluation: measurement of methylmalonic acid and homocysteine vs vitamin B (12) and folate’, Clinical Chemistry, 46, pp. 1277–1283. Available at: 10.1093/clinchem/46.8.1277 10926922

[5] Mazow, M. (1978) ‘The four-step test for diagnosis of paralytic and/or restrictive strabismus’, Ophthalmology, 86, pp. 1397–1400. Available at: 10.1016/S0161-6420(79)35381-7 542242

[6] Pianka, P., Almog, Y., Man, O., Goldstein, M., Sela, B.A. and Loewenstein, A. (2000) ‘Hyperhomocysteinemia in patients with nonarteritic anterior ischemic optic neuropathy, central retinal artery occlusion, and central retinal vein occlusion’, Ophthalmology, 107, pp. 1588–1592. Available at: 10.1016/S0161-6420(00)00181-0 10919914

[7] Sachdeva, V., Mittal, V., Pathengay, A., Kekunnaya, R., Gupta, A. and Rao, B.V. (2013) ‘Isolated abducens nerve palsy with hyperhomocysteinemia: association and outcomes’, Indian Journal of Ophthalmology, 61(10), pp. 598–600. Available at: 10.4103/0301-4738.121089 PMC3853462 24212315

[8] Sowbhagya, H.N., Kothari, M., Kumar, L.K. and Nikhil, N. (2015) ‘Ocular manifestations of hyperhomocysteinemia and their response to therapeutic modalities’, International Journal of Scientific Studies, 3(3), pp. 55–59. Available at: 10.17354/IJSS/2015/269

